# Introducing the Ko Corpus of Korean Mother–Child Interaction

**DOI:** 10.3389/fpsyg.2020.602623

**Published:** 2020-12-30

**Authors:** Eon-Suk Ko, Jinyoung Jo, Kyung-Woon On, Byoung-Tak Zhang

**Affiliations:** ^1^Department of English Language and Literature, Chosun University, Gwangju, South Korea; ^2^Department of Linguistics, University of California, Los Angeles, Los Angeles, CA, United States; ^3^Department of Computer Science and Engineering, Seoul National University, Seoul, South Korea

**Keywords:** Korean, corpus, CHILDES, Mean Length of Utterance, child-directed speech, adult-directed speech, familiarity

## Abstract

We describe a corpus of speech taking place between 30 Korean mother–child pairs, divided in three groups of Prelexical (*M* = 0;08), Early-Lexical (*M* = 1;02), and Advanced-Lexical (*M* = 2;03). In addition to the child-directed speech (CDS), this corpus includes two different formalities of adult-directed speech (ADS), i.e., family-directed ADS (ADS_Fam) and experimenter-directed ADS (ADS_Exp). Our analysis of the MLU in CDS, family-, and experimenter-directed ADS found significant differences between CDS and ADS_Fam, and between ADS_Fam and ADS_Exp, but not between CDS and ADS_Exp. Our finding suggests that researchers should pay more attention to controlling the level of formality in CDS and ADS when comparing the two registers for their speech characteristics. The corpus was transcribed in the CHAT format of the CHILDES system, so users can easily extract data related to verbal behavior in the mother–child interaction using the CLAN program of CHILDES.

## Introduction

Over the last several decades, there has been an increasing emphasis on the use of spontaneous speech data in the study of language. The importance of ecologically valid spontaneous speech data has particularly been viewed critical in the area of child language acquisition where research questions often involve biological predisposition to language development (Johnson et al., 2013; Soderstrom and Wittebolle, 2013; Cristia et al., 2019). Earlier studies of child language, especially those focusing on phonological aspects of language development, used to base their investigation on a handful amount of data collected in a lab setting (e.g., Stoel-Gammon and Buder, 2002). However, data collection with young children poses various challenges since it is hard to maintain their focus on the task and usually not feasible to elicit target words or constructions of interest from young participants in a lab setting. With the development of technology, increasingly more researchers are creating a corpus of spontaneous speech in a natural setting. Yet, transcribing vocalizations or speech produced by young children and even the adult when interacting with their children is quite challenging compared to the conventional lab speech or adult-to-adult conversation. As such, despite the importance of spontaneous data in the research of child language acquisition, it takes considerably more time and efforts to create a corpus involving young children and their caregivers compared to constructing a corpus of speech between adults. In this regard, making such data accessible via a repository such as the CHILDES database is important for the research community directly or indirectly working on issues related to language acquisition.

With the advancement of densely connected internet and affordable disk space on the one hand, and the movement toward the practice of open science in which researchers share their data and scripts with the community on the other, the amount of child language corpus downloadable for free on the internet has been on a rapid rise. Despite the many number of corpora available in the CHILDES database, however, there are very few corpora of Korean mothers interacting with their children publicly available yet. Further, there is very little information on the construction of mother-child interaction corpora, in particular a detailed description of the transcription criteria, despite the many decisions that have to be made against challenges posed by the nature of spontaneous speech. The Ko corpus of Korean mother–child interaction was created to help fill in this gap, and this paper provides detailed information on how this corpus was created and the transcription criteria along with the rationale for making judgments for a number of issues.

The present corpus is differentiated from the existing body of open-access child language data in several regards. First, the data collection was designed to include the adult-directed speech (ADS) as well as the child-directed speech (CDS) from the same speakers. Though there are other corpora which include occasional adult-to-adult conversations, our corpus was designed to include the ADS at the outset so that research comparing the registers of CDS and ADS can find the data more systematically. Further, there are two different formalities of ADS provided, i.e., the family-directed ADS (ADS_Fam) collected while the mother talks with her family member or a close friend over the phone, and the more formal experimenter-directed ADS (ADS_Exp) capturing the interaction of the mother with the experimenter. Most previous studies involving ADS elicited ADS_Exp simply by having the mother engage in a conversation with the experimenter (e.g., Bernstein Ratner, 1984; Buckler et al., 2018). In the literature of sociolinguistics, however, it is well-known that the level of formality in the relation between interlocutors results in differences in speaking styles (e.g., Labov, 1966, 1972; Maclagan, 2000). In this regard, it would be ideal to balance the formality between the CDS and ADS when comparing differences in the speech as a function of speech register which varies according to the developmental needs of the language learning listener. Since CDS is a speech register that is likely to be on the lowest end of the formality continuum, we sought to elicit a comparable formality of ADS by having the mother talk with one of her family members or close friends.

Second, we provide manually segmented crisp utterance boundaries so that research looking into temporal aspects of mother–child interaction can be conducted with more accuracy. The first step necessary for the preparation of the audio transcription is cutting up the long stream of recoding into a manageable sized sequences. The criterion we adopted in the creation of our transcription is to cut the utterances without including the pauses before or after the actual speech articulation. It seems that most of the corpora previously released for open access do not have a specific guideline as to where to draw the utterance boundaries. Thus, a piece of speech stream segmented as a single utterance would include a varying duration of silence either preceding or following the actual articulation of an utterance. For this reason, an utterance segment that might appear to be a 10 second-long utterance according to the time stamps may actually contain a speech articulation that lasts only for 2 seconds. Such a segmentation practice might not be relevant for many researchers but can cause a problem when the research question is related to temporal aspects of speech such as speaking rate (e.g., Ko, 2012) or response time in turn taking (e.g., Casillas et al., 2016). The transcription of our corpus includes an explicit interval for non-speech, usually the silence interval, included with time stamps as one of the main tiers (^∗^PAU) in addition to the usual speaker tiers such as the mother (^∗^MOT) and the child speech (^∗^CHI) tiers. With the adoption of an explicit segmentation scheme, research dealing with temporal aspects of mother–child interaction would benefit in drawing a more informative conclusion and approximating the actual numbers. For example, Ko (2012) analyzed the Providence corpus in the CHILDES and found that mothers’ speaking rate accelerates in the multi-word stage of the children. Her calculation of the speaking rate was based on the time stamps in the transcript thus margins before and after the utterances were also included in the duration of the utterances. Calculations based on such non-crisp boundaries should still yield a valid result when it comes to capturing the developmental tendency because the noise in the data was randomly distributed across all transcription. It was, however, hard to interpret the actual speaking rate yielded by this calculation due to the inclusion of silences in segmentating the utterances.

The current corpus serves as the only cross-sectional open-access multimodal corpus of mother-child spontaneous interaction in the Korean language as of this writing. There are currently two additional corpora of Korean available in CHILDES, the Jiwon^1^ and the Ryu (2012) corpus. The Ko corpus is differentiated from them in the following respects. First, it contains a relatively large number of participants with 30 mother-child dyads compared to Jiwon, which contains only one child, and the Ryu corpus, which contains three children. Second, it is a cross-sectional data set containing three groups of Prelexical, Early-Lexical, and Advanced-Lexical stages, which makes it possible to investigate the developmental characteristics of mother–child conversations at each stage of language acquisition based on a sizable number of children compared to typical longitudinal studies. In comparison, the Ryu corpus is a longitudinal data set, which can serve as a great resource for questions on developmental changes although on a limited number of children. Third, it provides a multimodal data set of spontaneous interactions between the mother and child.^2^ Jiwon is also spontaneous but it provides only the transcripts whereas Ryu is multimodal but the activity centers around book reading.

As mentioned earlier, very few corpus of child language provides detailed documentation about the construction of the corpus, which is understandable. The data collection is usually conducted to investigate a specific research question, thus the documentation is provided in the methods section of a paper delivering the research outcome based on the data. Also, the existence of an extensive guideline for transcription already available in the CHAT documentation (MacWhinney, 2000) of the CHILDES might make it seem redundant to provide a separate documentation on the transcription criteria. Nevertheless, corpus users may benefit from knowing the details of how the corpus was constructed beyond the information available in the methods section of the related research articles. This is the case for a corpus with its own conventions like ours, or a non-English corpus, which sometimes contains language-specific issues not thoroughly resolved according to the guidelines in the CHAT documentation.

In this paper, we provide a detailed description of the criteria involved in the construction of our corpus. In particular, we describe our decisions and detailed guidelines on the transcription. We then provide basic summary statistics of the corpus and statistical analysis of lexical and utterance-level characteristics of Korean CDS compared to ADS_Fam and ADS_Exp.

## Description of the Corpus

### Participants

We recruited a total of 36 infant–mother dyads, 12 in each of the three developmental stages of Prelexical, Early-Lexical, and Advanced-Lexical group (Table 1). The participants were recruited primarily through advertisements in on-line bulletin boards targeted for mothers living in Seoul and personal referrals. They were paid for participating in the experiment. A total of 35 dyads’ speech was transcribed after eliminating one recording from the Early-Lexical group due to the child’s excessive crying. Of these, 30 mothers consented to releasing the audio recording and accompanying transcripts to publicly available data repository. The audio and transcripts of these 30 mother–child interactions, 10 in each developmental stage group, are available in the CHILDES database^3^. We report statistics on both the full (*n* = 35) and the open-access (*n* = 30) data set here to serve the readers of our published articles based on the full data set as well as potential users who are looking to use the open-access data set.

**TABLE 1:** Number of children in each developmental group.

Group	Age		Sex	
		
	Original data (*n* = 35)	Open-access (*n* = 30)	Original data (*n* = 35)	Open-access (*n* = 30)
Prelexical	0;6.2 - 0;9.23 (*M* = 8 months, SD = 40 days)	0;6.2 - 0;9.23 (*M* = 8 months, SD = 44 days)	8 Males, 4 Females	6 Males, 4 Females
Early-Lexical	0;11.14 - 1;4.3 (*M* = 14 months, SD = 40 days)	1;0.15 - 1;4.3 (*M* = 14 months, SD = 35 days)	6 Males, 6 Females	4 Males, 6 Females
Advanced-Lexical	2;1.10 - 2;6.24 (*M* = 27 months, SD = 49 days)	2;1.13 - 2;6.24 (*M* = 27 months, SD = 51 days)	8 Males, 4 Females	6 Males, 4 Females

The mean age of the children in each of the developmental groups was 8, 14, and 27 months, respectively. Note that the age interval between the three groups is not equal, i.e., there is a 6-month gap between the Prelexical and the Early-Lexical group, but a much greater gap of 13 months between the Early-Lexical and the Advanced-Lexical group. This is because the target age in each group was set suitable to investigate questions across specific developmental stages. Infants in the Prelexical stage cannot yet produce words but are able to vocalize syllables. Infants in the Early-Lexical stage are just beginning to produce their first word though their speech is not yet fully intelligible. Mothers are known to adapt their speech on observing the production of their child’s first speech around this time (Phillips, 1973; Snow, 1977; Ko, 2012; Matsuda et al., 2014). Children in the Advanced-Lexical stage have accumulated almost a thousand words in their lexicon, and can put them together in an utterance containing quite a long string of words. The rapidly changing linguistic maturity during the first 3 years of life can thus be compared with data from these distinct developmental stages.

The participating mothers all spoke Korean as their native language, and were overall quite highly educated. They were all recruited in Seoul and spoke Seoul dialects except one mother who had a Kyungsang accent. Their educational background included 2 high school graduates, 23 college degrees, and 11 graduate level education. Nationwide, about 69.7% of high school graduates are reported to go to college as of 2018 according to the Statistical Yearbook of Education published by the Ministry of Education in Korea. Thus our participants have an educational level higher than the national average. Their mean age was 33, and ranged from 28 to 41.

### Data Collection Environment

**FIGURE 1:**
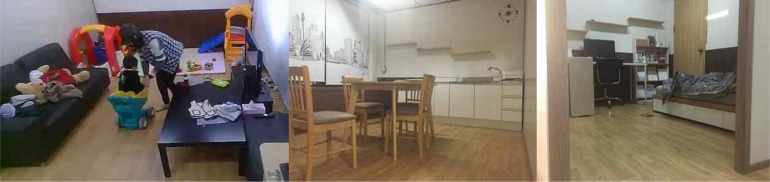
The pictures show the environment of the apartment where the data were collected. The living room on the left was equipped with age-appropriate toys. The kitchen in the middle was equipped with a fridge and a kitchen table with chairs. The bedroom on the right had a bed, a desk, and a bookcase. Not shown is a foyer where participants took off their shoes and unused toys were stored.

Data were collected at a space set up in the Biointelligence Lab of the Computer Science Department in Seoul National University, which was essentially a mock apartment intentionally designed to simulate the natural home environment as closely as possible with its structure and the furnishing (Figure 1). The apartment consisted of a foyer with space to take shoes off, a living room with a sofa and a table as well as toys and books appropriate for the age of the participating children, a bedroom furnished with a bed, a desk, and a bookcase, and a kitchen equipped with a fridge and a kitchen table with chairs. Participants were not given any particular instructions as to which space to use, but most of them naturally spent the majority of the time in the living room where toys were furnished.

### Procedure

The infant–mother dyads were greeted with the second and the third author. After a brief explanation about the recording and clarifying any questions, the mothers signed the consent forms. The mother and the child were then fitted with vests equipped with a clip-on microphone wired into a digital recorder in the pocket. Before the recording session began, we made a sharp hand clap noise to be used for synchronizing the signals on all devices involved. The recording was made with a 44.1-kHz sampling rate, 16 bits per sample in the wav format. The two channels of recording, one from the mother and the other from the child, were later time synchronized and merged to form a file with a stereo channel. The mother also wore a cap with a clip-on video-camera to record the first-person view of the scene, and two wrist-worn devices with various types of physiological sensors.

Mothers were told to interact with their infants as they would normally do at home, making use of the toys and books at their will. They were then left alone to be engaged in a free-play session for 40 min. One of the researchers monitored the video-recording of the free-play session in a separate room via multiple wireless cameras. When the 40 min have passed, the researcher made a phone call to the mother from an adjacent room, and asked to make a phone call to one of her family members or close friends and talk for about 5 min. They were already informed of this step before they visited for recording, so had already arranged someone to call. The purpose of this second task was to elicit ADS_Fam. As noted above, many similar studies simply get the researcher interact with the mother but we conducted this ADS session with someone who is already familiar with the mother so that a level of formality comparable to the mothers’ CDS could be achieved for the ADS data. In a few cases where the father came along but was instructed to wait outside, we let him enter the lab and chat with the mother in person for a few minutes. The researcher then came in and talked with the mother for a few minutes. This last interaction activity yielded ADS_Exp, which differs from ADS_Fam in the degree of formality thus forms a separate speech register.

### Assessment of Language Development

Mothers were sent a booklet for assessing the infants’ language development via mail before they visited for the recording along with the documents explaining the experiment and the agreement form to participate. The mothers responded to Sequenced Language Scale for Infants (SELSI, Kim et al., 2003), a parent-report instrument for assessing 4- to 35-month-old infants’ receptive and expressive language abilities. Among the participants, the scores of two children in the receptive, and five in the expressive test indicated a slight deviation from the norm. Further, one in the receptive and three children in the expressive language test received a score indicating language delay. However, among the three delayed children in the expressive test, two were ages 6 and 7 months old, respectively, for whom assessments of communicative developments by parental report are prone to error due to the very small number of vocabulary that they understand at this age. The third indicated a normal developmental score in the receptive language test.

## Transcription

The interactions between mothers and their children were transcribed according to the CHAT transcription format (MacWhinney, 2000) with some simplifications. A good part of our transcription conventions are, therefore, reiterations of the guidelines in the CHAT manual. We, however, repeat them here despite the overlap with the standard CHAT conventions, so that readers can quickly zoom in on all and only the relevant set of conventions specific to constructing the corpus of our kind, i.e., multimodal spontaneous interaction of Korean mother-child dyads. In describing our transcription guidelines below, comments are made where we diverge from the conventions suggested in MacWhinney (2000). A total of 10 undergraduate and graduate students who are native speakers of Korean participated in the initial transcription process. They were trained by the second author until they indicated a clear understanding of the guidelines provided below. They then transcribed the recordings by listening to the audio recordings using the interface provided in the CLAN program. All the transcripts were second-passed by the second author to ensure quality and consistency. Video recordings were also consulted in this process when contextual information was necessary for clarification of the context. In the open-access transcript set, children’s names were replaced by the word NAME to deidentify the participants.

### Utterance

**FIGURE 2:**
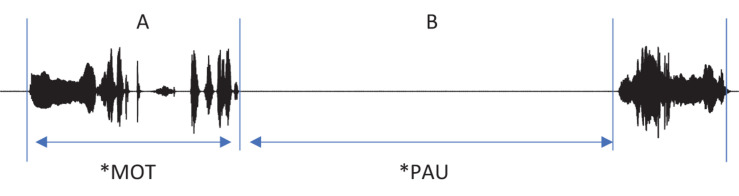
Segmentation of utterances. In the interval **(A)**, there are more than one sentences produced without a pause. These were segmented as a single utterance, and the speaker was indicated in the main tier, the mother (*MOT) in this example. In the interval **(B)**, the non-speech portion of the recording between utterances was indicated as *PAU on the main tier.

Each main line of the transcript consists of one utterance produced by one participant. According to CHAT, there must be only one of the three utterance terminators (period, question mark, and exclamation mark) on each line. Commas can be used as many times as needed on a line in order to indicate phrasal junctures. Each utterance usually consists of one sentence. However, when a speaker produced one sentence right after another without a perceptible utterance boundary, the two sentences were considered to comprise one utterance and thus transcribed on the same line (Figure 2, Interval **A**). While it should be acknowledged that each transcriber might have different judgments on whether two sentences form one utterance or two, all the transcripts went through a second pass by the second author for consistency across transcripts. In this way, we minimized any discrepancies on judging the utterance boundary among transcribers. All utterances in the transcript have time stamps for the beginning and the end of the utterance in the corresponding audio file so that they can be extracted for acoustic analysis.

### Main Tiers

A transcript mainly consists of the main tiers and the dependent tiers. A main tier shows the basic transcription of speakers’ utterances and always starts with a participant ID. The participants include the child (^∗^CHI), the mother (^∗^MOT), and adult researchers (ADU, ^∗^AD1, ^∗^AD2, ^∗^AD3). It is usually the case that two researchers (^∗^AD1 and ^∗^AD2) took care of a recording session with one exception in which only one researcher (^∗^ADU) appeared. Occasionally, the child’s father (^∗^FAT) comes into the room to talk with the mother after the 40-min free-play time, as mentioned earlier. The remaining parts of the recording where none of the participants speaks are tagged with ^∗^PAU, e.g., when there is silence or any non-human speech sounds (Figure 2, Interval **B**). In other words, ^∗^PAU fills the intervals between human utterances. ^∗^PAU is always transcribed with “0,” except when indicating the beginning and ending of each CDS and ADS session (see “Register Blocks” for details). Though ^∗^PAU is not a speaker tier, we introduced this tier for research dealing with temporal aspects of the speech signal. Whereas most other corpora would just include the pause at the end of the first utterance, we demarcated the non-speech interval with two time stamps to facilitate a more accurate estimation of phonetic variables such as speaking rate.

### Dependent Tiers

Dependent tiers provide additional coding for further information. Among a number of dependent tiers suggested in the CHAT transcription format, those employed in our transcripts are the orthography tier (%ort), the gloss tier (%gls), the comment tier (%com), and the explanation tier (%exp). The orthography tier provides transliteration of Korean to Roman letters. The gloss tier provides the gloss of children’s innovative forms. The comment tier was mainly used to provide English orthography for English words whose pronunciation was transcribed in Korean in the main tier. The explanation tier is used to provide any extra explanation about the utterance. Further explanations for each of these dependent tiers are provided below.

**%ort**: The transcription of our corpus was done in Hangul, the Korean alphabet. However, we added the Roman transliteration in the %ort tier for those who might find the inclusion of this tier to be useful. There are several different standards for Korean-to-Roman transliteration, but we followed the Yale romanization convention. The transliteration does not reflect phonological changes but is a one-to-one mapping of the Korean letters to a Roman alphabet except for some cases such as the empty onset in the syllable initial position as directed by the Yale Romanization convention. Since it is a dependent tier, a CLAN command such as *freq* on the %ort tier will yield results with information about the speaker on its main tier.

**%gls**: Gloss tier (%gls) provides a translation of the child’s speech into the adult language. Even when the child’s speech itself is hard to interpret, the transcriber could understand its meaning by referring to the mother’s responses and the context in which it was uttered. Whenever needed, video recording was also consulted to infer the meaning of child-invented forms. Note that the translation provided in italic in the following examples is not present in the transcripts.


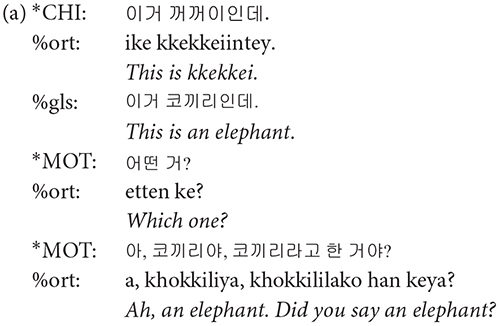


**%com**: English loanwords account for 4.5% of the word type, and 1.4% of word tokens in the colloquial speech of college students (Im and Ahn, 2004). With increasing exposure to English and global experiences, gradually more Korean speakers are adopting Korean-English code switching. Such tendency was clear among our participating mothers, the young urban females often reported to lead sociolinguistic changes (Labov, 2001; Eckert and McConnell-Ginet, 2003). When a speaker produced words or phrases in English, we transcribed them in Korean as actually pronounced in the main tier, with the English orthography in %com tier. The comment tier was employed only when the speakers were considered to have “chosen” to speak in English, e.g., when a mother was teaching an English word to her child. English loanwords that are lexicalized in Korean were not accompanied by its English orthography in the comment tier. For example, established loan words such as *computer* and *juice* were simply transcribed in Korean in the main tier without further annotating an English orthography in a %com tier. Some of the examples when we did add the annotation in the %com tier are illustrated below. In the second example, the angle brackets < > indicate that all the words in the brackets are the speaker’s singing; if there were no brackets, [=! sings] would mean that only the word that immediately precedes it is part of the speaker’s singing.


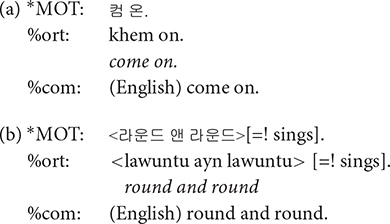


**%exp**: The explanation tier is useful for specifying the referent of a noun or a deictic identity of objects that can be inferred from the context. In the open access transcript, we do not provide an English translation for this tier.


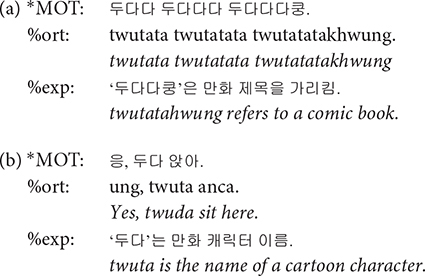


### Register Blocks

As mentioned earlier, each recording session consisted of three blocks, i.e., CDS, ADS_Fam with an intimate person, and ADS_Exp with the experimenter. In the first ADS, or the ADS_Fam block, mothers were asked to talk to an adult close to them, mostly her mother or husband. In the second ADS, or the ADS_Exp block, mothers interacted with experimenters who were not familiar to them. In the transcripts, the beginning and end of each block is marked on the ^∗^PAU tier with the following notations: “cds starts,” “cds ends,” “first ads starts,” “first ads ends,” “second ads starts,” “second ads ends.” Based on these markings, it was expected that each utterance could be categorized into CDS or different speech registers of ADS. However, even within a CDS block, mothers occasionally spoke to adults when, for example, receiving a phone call. Also, mothers often talked to their infants while interacting with researchers. We therefore tagged each adult-directed utterance, regardless of whether it occurs in CDS or ADS block, with “ads-fam” for speech directed to intimate adults and “ads-exp” for speech directed to experimenters.

### Coding Conventions and Symbols

Below we introduce special form markers used in our corpus. They were largely adopted from the CHAT convention, but some of them were simplified.

**(..)**: Pauses are coded with symbol (..). The CHAT convention distinguishes (..) and (…) depending on the length of the pause, but in our data we only used (..) regardless of how long the silence is. Even when there is a long pause within an utterance, it is transcribed in one line, rather than entering the pre-pause and post-pause material on separate lines. In such cases, transcribers were able to judge the speaker’s intention to continue after the pause based on intonational cues.


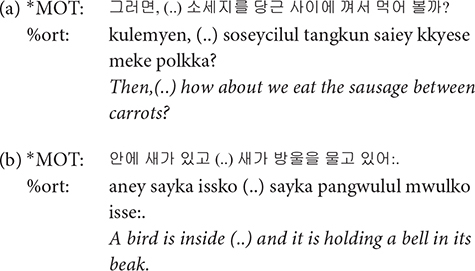


**[: text]**: The data being spontaneous speech between two intimate speakers, the conversation includes a great deal of informal speech. We transcribed the non-standard casual form as it is actually pronounced, along with its standard form as listed in the dictionary in square brackets that follow. It should be noted that even when a casual form was used, we did not annotate its standard counterpart when the casual form is registered as an entry in the dictionary. For example, 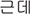 *kuntey* is not followed by its standard form 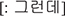, because it is listed in the dictionary.


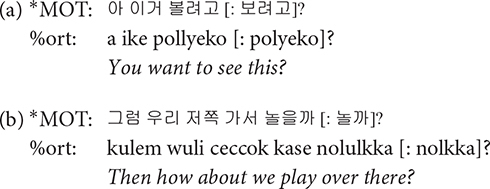



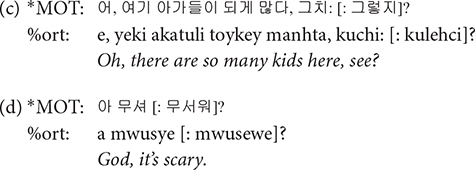


**[/]**: The [/] symbol is used for what is referred to as repetition and retracing in CHAT transcription format. CHAT format provides distinct notations for repetition, i.e., saying something and repeating it without any change, and retracing, i.e., repeating some material with different formulation while maintaining the same idea, but we coded both repetition and retracing invariably with [/]. It is transcribed following the material that is being repeated or retraced. When two or more words are being repeated or retraced, they are enclosed in angle brackets, as can be seen in the third example below.


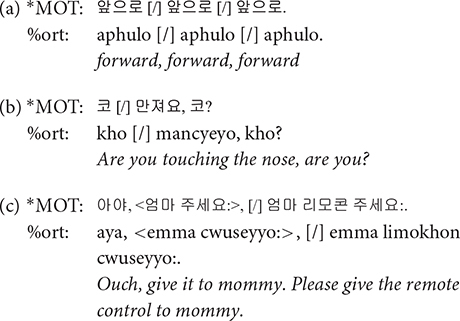


**xxx:** The symbol xxx is used for unintelligible speech with unclear phonetic shape due to various reasons such as poor recording qualities and overlapping with background noise. It is also used when speakers make some weird noises that cannot be transcribed in human language, e.g., screaming and gasping. It should be noted that xxx symbol can only be used when the phonetic shape of the material is unclear. When it is possible to identify the phonetic shape of a word but its meaning is uncertain, @u should be used.

**@u**: A word is tagged with @u when its pronunciation is clear but the meaning is unclear. This symbol can be used to tag words produced by children that seem to bear some meaning, which is unintelligible. It differs from @b in that @b is attached to babbling words that do not normally have specific lexical meaning. Sometimes even the words produced by mothers are tagged with @u. The material for which gloss tier (%gls) provides its meaning does not accompany @u in the main tier, as its meaning is clear.


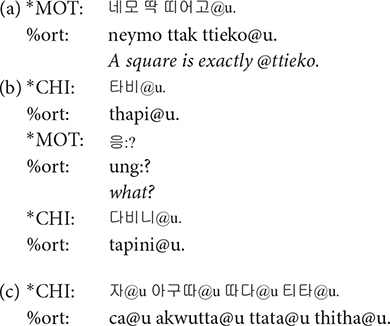


**@b**: The @b symbol is used for babbling words that do not seem to carry any lexical meaning. @b is used almost exclusively for infants in the Prelexical group.


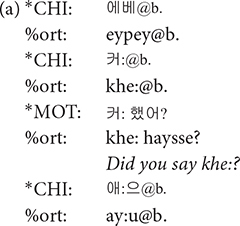


**@wp**: A word containing word play was tagged with @wp symbol. Words were judged as containing word play when the prosody was significantly modified resulting in unusually low or high pitch or rapidly changing syllable durations and thus differ from usual conversational speech. Typical examples of word play include rhythmical word repetitions and chanting, as in (a) (the use of @z:owp will be discussed shortly), similar to the patty cake game in the western culture, and cases where mothers change their voice in order to produce onomatopoeic sounds as in (b). Word play also includes producing nonsense words with playful vocalization mainly to amuse the children and grab their attention, as shown in (c).


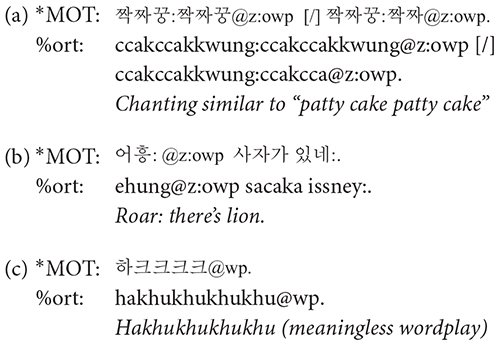


**@o**: Korean has a rich inventory of ideophonic words, describing either auditory (analogous to onomatopoeia in English) or non-auditory experiences. Ideophonic expressions were marked with @o symbol. The tagging of @o was based on the dictionary definition of ideophones, i.e., a word that depicts the way something is done or the sound something makes. In many cases, ideophonic words accompanied word play, when, for example, the speaker imitates the sound of an animal or an object. We coded such words with @z:owp, employing @z of the CHAT convention that marks user-defined special forms, as shown in (a) and (b) above.

**: :** A colon was used to mark lengthened syllables as judged notable by perception. Syllables were often lengthened in expressive words such as onomatopoetic words and interjections.


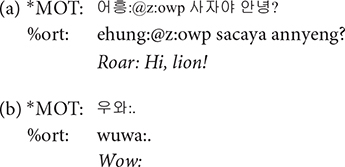


An alternative way of transcribing an elongated word-medial vowel would be to transcribe the vowel multiple times. For instance, for the onomatopoeic word 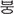 *pung* which depicts the sound of a driving car, a comic book might transcribe such an instance as 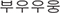 *puuung* or 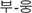 *pu-ung*. We simply noted the elongation by adding the : symbol on the right edge of the syllable as in 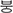 : *pung*:.

**&**: The & symbol is attached to the beginning of a phonological fragment or a lexical item that can be regarded as a false start. Note that a word or a fragment preceded by this symbol is omitted in calculating the MLU in CLAN.


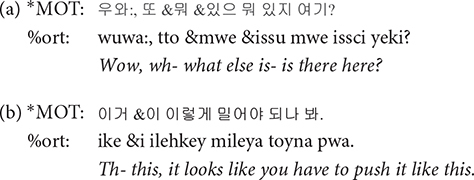


**&=text**: Non-linguistic sounds are described using &=symbol. One of the most common use of this notation in our data is in & = laughs.


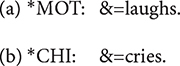


**[=! text]**: Paralinguistic effects that arise during speech is noted using [=! text]. We developed three types of this notation for singing, reading, and role-playing. Only the part of an utterance enclosed by angle brackets is accompanied by the event noted in the square brackets.


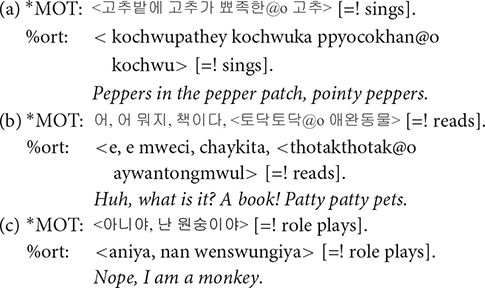


### Overlapping Speech

We did not adopt any particular notation for indicating overlapping speech. When two or more utterances produced by different speakers overlap, the one whose beginning time is earlier is transcribed preceding the one with a later beginning time.

Under the principle that one utterance be transcribed on one main line, even an utterance that is interrupted or broken up by the conversational interactions or back-channel signals of another speaker is entered on the same line as in (a), not (b). One may argue that these back-channel signals do not constitute turns but simply indicates the listening of the interlocutor, but they also provide an indication that the listener would like the speaker to continue (Macaulay, 2006). Our rationale for choosing (a) was that it can capture the fact that the child produced the Advanced-Lexical utterance. It was often possible to judge by intonational patterns whether the child intended to produce a string of words as one utterance.


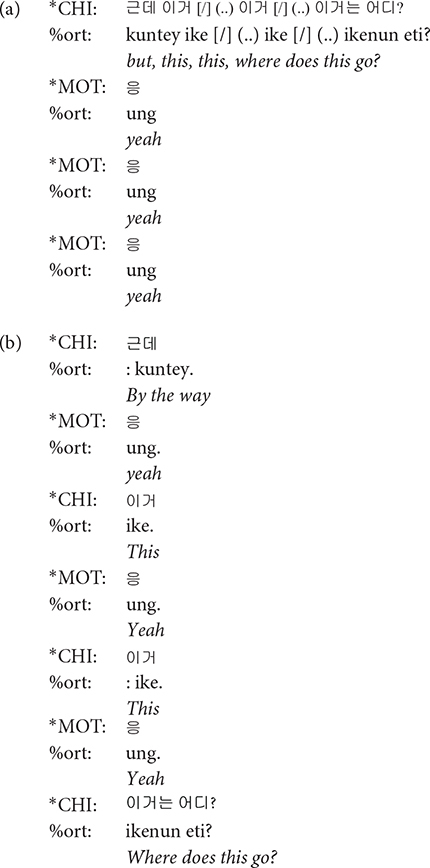


### Orthographic Issues: Spacing and Non-Korean Words

We followed the conventions of Korean orthography as set forth by the National Institute of Korean Language (2017). In particular, special care was taken to maintain a space before a dependent noun as in (a) and an auxiliary verb as in (b). These are prosodic groups with the previous word as a single unit, thus the spacing is often overlooked in casually written texts.


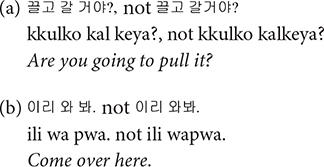


## Basic Corpus Analysis

We present some basic distributional statistics of the corpus to show the lexical and utterance-level characteristics of Korean mothers’ child-directed speech. We then present statistical analyses for some of the lexical and utterance-level characteristics. The individual speakers’ data for the word tokens, word types, utterances, and duration used for the statistical analysis can be found in the Supplementary Data Section.

### Corpus Statistics

We first provide each group’s total number of word tokens and the number of utterances in each register. Note that the number of words and utterances can vary depending on how you calculated them. For example, one might exclude an unintelligible word transcribed as *xxx* or an utterance entirely composed of sounds tagged as false start or repetition, with the *&* or the *[/]* symbol, from the number of word tokens or the utterances. Depending on the purpose of the analysis, however, one might choose to include those irregular vocalizations as part of the utterance as in calculating the utterance duration or gap duration between taking turns in conversation, and so on. This is why the output of the clan command timedur, for example, may yield a greater number of words and utterances than mlu though an option can be added to include or exclude the irregular words. The numbers we provide in the Tables 2, 3 are based on the mlu and freq functions, which yield a more conservative number than timedur by excluding irregular vocalizations and repetitions from counting. Note that the total number of word types in Table 2 is smaller than the sum of the word types in the three groups due to the overlap in word types across the groups.

**TABLE 2:** Number of word tokens and types in the original Ko corpus. (Numbers in parentheses are for the open-access data).

	Prelexical	Early- Lexical	Advanced-Lexical	Total
Total number of word tokens	23,142 (19,789)	24,634 (21,481)	28,696 (23,694)	76,472 (64,964)
Number of tokens in CDS	18,867 (16,028)	19,755 (17,986)	23,489 (19,093)	62,111 (53,107)
Number of types in CDS	4,230 (3,898)	4,027 (3,720)	4,649 (4,111)	9,270 (8,526)

**TABLE 3:** Number of utterances in the original Ko corpus. (Numbers in parentheses are for the open-access data).

	Prelexical	Early-Lexical	Advanced-Lexical	Total
CDS	7,113 (6,039)	7,267 (6,717)	7,637 (6,206)	22,017 (18,962)
ADS_Fam	608 (490)	455 (336)	592 (480)	1,655 (1,306)
ADS_Exp	183 (165)	403 (217)	364 (282)	950 (664)

### Lexical Characteristics

**FIGURE 3:**
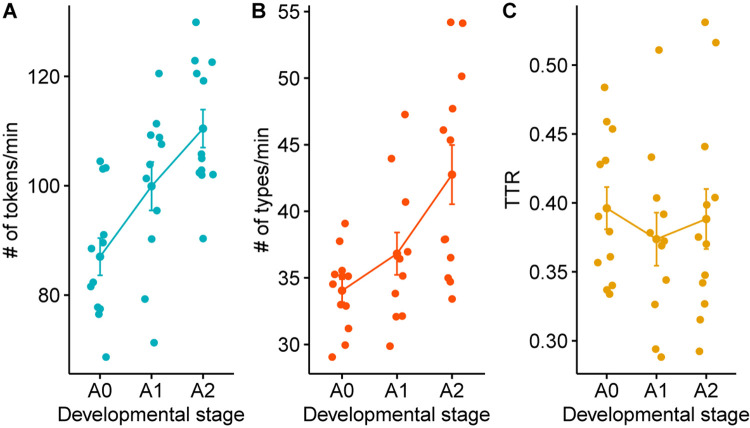
Lexical characteristics across developmental stages. The x-labels A0, A1, and A2 refer to the developmental stage of Prelexical, Early-Lexical, and Advanced-Lexical stage, respectively.

We calculated the number of word tokens, types, and type-token ratio of the CDS in our data by using the freq command in CLAN. Since each transcript contains a varying amount of recording time, we cannot directly compare the number of tokens based on the output of the freq command. In order to normalize the number of tokens and types based on the amount of time speech was produced in each session, we calculated the entire duration of CDS utterances excluding the between-utterance pauses using the timedur command. We then compared the number of word tokens and types in each child group by dividing them by the accumulated utterance duration. As can be seen in Figure 3, there was a steady increase in the number of tokens and types as the child’s language developed. A one-way ANOVA using the aov function of R (R Core Team, 2019) showed an increasing amount of word tokens [*F*(2,32) = 10.04, *p* < 0.001] and types [*F*(2,32) = 7.35, *p* < 0.01] with child age. A computation of Tukey HSD (Tukey Honest Significant Differences, R function: TukeyHSD()) for performing multiple pairwise-comparison between the means of developmental groups found a significant difference in tokens (*p* < 0.001) and types (*p* < 0.001) between the Prelexical and the Advanced-Lexical group, a marginally significant difference in tokens (*p* = 0.055) but not in types (*p* = 0.48) between the Prelexical and the Early-Lexical group, and no significant difference in tokens (*p* = 0.13) but in types (*p* < 0.05) between Early-Lexical and Advanced-Lexical group. The type-token ratio (TTR), a measure of lexical diversity, however, did not show a statistically significant pattern [*F*(2,32) = 0.35, *p* = 0.7] The absence of an effect in the TTR across groups, however, might not necessarily indicate lack of developmental changes in lexical diversity. Though TTR has often been considered as a measure of lexical diversity (Carroll, 1938; Fletcher, 1985), there are studies suggesting a more nuanced interpretation of the TTR due to its susceptibility to the word classes (Richards, 1987), sample size, and the contexts (Montag et al., 2018). Further, given the agglutinative nature of Korean morphology, it is an issue that would benefit from a more in-depth investigation.

One of the notable characteristics of the CDS at the lexical level at this stage of language development is the frequent use of onomatopoeic words. Children beginning to learn words work hard on mapping form to meaning, whose arbitrary relation is considered one of the hallmarks of human language (Hockett, 1973) but is a challenge for word learning. It has long been considered that the close resemblance between form and meaning in sound symbolic expressions might make it easier for children to tackle the task of word learning (Farrar, 1883; Berko-Gleason, 2005; Imai and Kita, 2014). In Jo and Ko (2018), we calculated the frequency of sound symbolic words including onomatopoeia, tagged with @o, expressive lengthening, tagged with :, and word play, tagged with @wp. We found that Korean mothers use expressive lengthening, onomatopoeia, and word play more frequently in the younger infants group. In addition, based on acoustic analyses of the associated audio, we found that mothers maintain acoustic saliency for sound symbolic words until later in development, although they somewhat weaken the prominence for ideophones when the child reaches a certain age.

### Utterance-Level Characteristics

Mean Length of Utterance (MLU) is considered to reflect syntactic complexity, and has often been used to represent child’s syntactic development. In general, mothers’ MLU gets longer with the child’s linguistic development (Ko, 2012). However, not much is known how it compares to the MLU of ADS. More specifically, ADS is further split into formal and informal speech registers depending on the addressee, but previous research usually overlooked the possibility that mothers’ ADS might vary in formality depending on the addressee (cf. Johnson et al., 2013). In order to compare mother’s MLU as a function of the child’s developmental stage and different speech registers, we divided mothers’ utterances into CDS, ADS_Fam and ADS_Exp using command-line perl functions. We then calculated the MLU, using the mlu command in CLAN, by dividing the total number of words by the number of utterances produced by each mother (Table 4).

**TABLE 4:** Mean Length of Utterances in words and Standard Deviation in the original Ko corpus. (Numbers in parentheses are for the open-access data).

	Prelexical	Early-Lexical	Advanced-Lexical	Total
CDS	2.53, 1.85 (2.51, 1.85)	2.59, 1.86 (2.55, 1.84)	2.98, 2.23 (2.97, 2.23)	2.71, 2.00 (2.68, 1.99)
ADS_Fam	4.61, 3.91 (4.71, 3.93)	5.23, 4.48 (5.62, 5.19)	5.12, 4.34 (5.20, 4.41)	4.96, 4.23 (5.12, 4.47)
ADS_Exp	3.57, 3.33 (3.75, 3.37)	3.49, 3.56 (3.29, 3.25)	2.94, 2.9 (3.07, 2.88)	3.30, 3.29 (3.31, 3.14)

**FIGURE 4:**
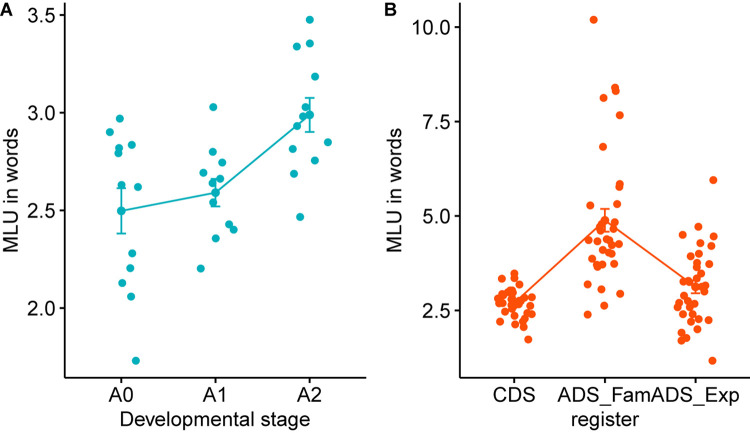
Mean Length of Utterance (MLU) across developmental stages **(A)** and speech registers **(B)**. The x-labels A0, A1, and A2 refer to the developmental stage of Prelexical, Early-Lexical, and Advanced-Lexical stage, respectively.

Mean Length of Utterance continues to rise throughout the children’s developmental stages from Prelexical to Advanced-Lexical, as shown in Figure 4. This indicates that utterances produced by mothers speaking to older children are longer and perhaps more complex, as measured by the number of words contained in each utterance. In particular, there is a greater increase in MLU as children progress from Early-Lexical to Advanced-Lexical stage. Note that Korean morphological system is classified as being agglutinative where a word commonly consists of multiple morphemes. Thus, the increase in MLU in our data might reflect a different degree of advancement in syntax than in a morpheme-based, or even word-based MLU of a non-agglutinative language such as English. A one-way ANOVA for the MLU in the three developmental groups found a significant main effect of Developmental Stage [*F*(2,32) = 7.81, *p* < 0.01]. A Tukey HSD found significant differences between Prelexical and the Advanced-Lexical (*p* < 0.01), and the Early-Lexical and Advanced-Lexical group (*p* < 0.05), but there was no significant difference between the Prelexical and the Early-Lexical group (*p* = 0.7).

We further compared the MLU between the three different registers of CDS, ADS_Fam, and ADS_Exp. A one-way ANOVA for the MLU in the three different registers showed a significant main effect of Register [*F*(2,102) = 32.45, *p* < 0.001]. A Tukey HSD found significant differences between CDS and ADS_Fam (*p* < 0.001), and ADS_Fam and ADS_Exp (*p* < 0.001). We, however, did not find any significant difference in the MLU of CDS and ADS_Exp (*p* = 0.302). This finding about the effect of registers involving the formality as well as the age of the addressee is one of the first in the literature of CDS, and suggests that it is ideal to match up the level of formality in CDS and ADS when comparing their patterns. In sociolinguistics, stylistic variation, whereby the same speaker expresses the same idea differently when addressing listeners with different level of familiarity, has been a classical topic of investigation since Labov (1972). Our results suggest that register effect of CDS, arising from the linguistic, cognitive, and developmental differences of the child addressee, needs to be separated from the differences that arise from stylistic variation depending on the degree of familiarity and formality between the speaker and the addressee.

## Conclusion

In this paper, we described the process of constructing the Ko corpus of Korean mother-child interaction in detail, and, in particular, presented a detailed guidelines for transcribing spontaneous speech of Korean mothers and young children. It is one of the few cross-sectional multimodal corpora available for Korean, and is also unique in that we segmented utterance boundaries as accurately as possible and that it has two different formalities of adult-directed speech included. Our analysis of the MLU in CDS, family-directed ADS, and experimenter-directed ADS found significant differences between CDS and ADS_Fam, and ADS_Fam and ADS_Exp, but not between CDS and ADS_Exp. Our finding suggests that researchers should pay more attention to controlling the level of formality in CDS and ADS when comparing the two registers for their speech characteristics. The corpus was transcribed in the CHAT format, so users can easily extract data related to verbal behavior in the mother-child interaction using the CLAN program of CHILDES (MacWhinney, 2000). The availability of this corpus is expected to facilitate research in child language acquisition and yield many new discoveries for researchers in various fields.

## Data Availability Statement

The datasets presented in this study can be found in online repositories. The names of the repository/repositories and accession number(s) can be found in the article/Supplementary Material.

## Ethics Statement

The studies involving human participants were reviewed and approved by the Institutional Review Board of Seoul National University. Written informed consent to participate in this study was provided by the participants’ legal guardian/next of kin.

## Author Contributions

EK conceptualized and directed the study. JJ and KO collected data. KO and BZ set up the recording environment and processed data for curation. EK and JJ set up the transcription criteria. JJ trained and supervised the transcribers. EK wrote the manuscript with contribution from JJ. All authors contributed to the article and approved the submitted version.

## Conflict of Interest

The authors declare that the research was conducted in the absence of any commercial or financial relationships that could be construed as a potential conflict of interest.

## Footnotes

1 https://childes.talkbank.org/access/EastAsian/Korean/Jiwon.html

2 As a reviewer points out, our corpus data might fall short of being representative of a child’s daily language experience since it involves playing in a new location in a confined setting. We have collected a complementary sort of data with the LENA system, a subset of whose derived data is being made available in an open repository (https://osf.io/uztxr/). Note, however, that every single syllable in the transcription is replaced with an X to render the content opaque. This was to protect the privacy of the family so the sort of analysis that one can conduct with them is different from what one would usually do with the current set of transcribed data.

3 https://childes.talkbank.org/access/EastAsian/Korean/Ko.html
